# Altered Colonic Mucosal Polyunsaturated Fatty Acid (PUFA) Derived Lipid Mediators in Ulcerative Colitis: New Insight into Relationship with Disease Activity and Pathophysiology

**DOI:** 10.1371/journal.pone.0076532

**Published:** 2013-10-18

**Authors:** Mojgan Masoodi, Daniel S. Pearl, Michael Eiden, Janis K. Shute, James F. Brown, Philip C. Calder, Timothy M. Trebble

**Affiliations:** 1 Nestlé Institute of Health Sciences, Lausanne, Switzerland; 2 Medical Research Council, Cambridge, Cambridgeshire, United Kingdom; 3 Department of Nutritional Sciences, University of Toronto, Ontario, Canada; 4 Department of Gastroenterology, Portsmouth Hospital NHS Trust, Portsmouth, Hampshire, United Kingdom; 5 Institute of Biomedical and Biomolecular Sciences, University of Portsmouth, Portsmouth, Hampshire, United Kingdom; 6 Department of Gastroenterology, Taunton and Somerset NHS Foundation Trust, Taunton, Somerset, United Kingdom; 7 Human Development and Health Academic Unit, University of Southampton, Southampton, United Kingdom; 8 NIHR Biomedical Research Centre in Nutrition, University Hospitals Southampton NHS Foundation Trust and University of Southampton, Southampton, United Kingdom; Juntendo University School of Medicine, Japan

## Abstract

**Objectives:**

Ulcerative colitis (UC) is a relapsing inflammatory disorder of unconfirmed aetiology, variable severity and clinical course, characterised by progressive histological inflammation and with elevation of eicosanoids which have a known pathophysiological role in inflammation. Therapeutic interventions targetting eicosanoids (5-aminosalicylates (ASA)) are effective first line and adjunctive treatments in mild-moderate UC for achieving and sustaining clinical remission. However, the variable clinical response to 5-ASA and frequent deterioration in response to cyclo-oxygenase (COX) inhibitors, has prompted an in depth simultaneous evaluation of multiple lipid mediators (including eicosanoids) within the inflammatory milieu in UC. We hypothesised that severity of inflammation is associated with alteration of lipid mediators, in relapsing UC.

**Design:**

Study was case-control design. Mucosal lipid mediators were determined by LC-MS/MS lipidomics analysis on mucosal biopsies taken from patients attending outpatients with relapsing UC. Univariate and multivariate statistical analyses were used to investigate the association of mucosal lipid mediators, with the disease state and severity graded histologically.

**Results:**

Levels of PGE_2_, PGD_2_, TXB_2_, 5-HETE, 11-HETE, 12-HETE and 15-HETE are significantly elevated in inflamed mucosa and correlate with severity of inflammation, determined using validated histological scoring systems.

**Conclusions:**

Our approach of capturing inflammatory mediator signature at different stages of UC by combining comprehensive lipidomics analysis and computational modelling could be used to classify and predict mild-moderate inflammation; however, predictive index is diminished in severe inflammation. This new technical approach could be developed to tailor drug treatments to patients with active UC, based on the mucosal lipid mediator profile.

## Introduction

Ulcerative colitis (UC) is a chronic, relapsing intestinal inflammatory disorder of the colonic mucosa, with variable distribution but limited to the distal bowel (distal colitis and proctitis) in 60% of cases [1]. In distal colitis there is commonly a clear demarcation between inflamed and non-inflamed tissues that demonstrate contrasting patterns of immunomodulator release [2].

Despite advances in medical treatments, including biologics that target cytokine-led inflammatory responses for severe disease, long term control of UC is variable with available therapeutic interventions [3]–[5], with 57% of all patients following a relapsing and remitting clinical course [6], [7]. In mild to moderate relapsing disease only, a limited therapeutic repertoire is available to patients principally as oral or topical 5-aminosalicylic acid (5-ASA) and corticosteroids.

Mucosal inflammation in UC is characterised by an infiltrate of neutrophils, plasma cells and eosinophils, which correlate with disease severity and are predictors of disease relapse [8]–[10]. It is proposed that lipid inflammatory mediators, including eicosanoids, which are rapidly and locally formed and degraded in-situ, promote neutrophil chemotaxis [11], [12], a pivotal step in the inflammatory cascade. Furthermore, mucosal inflammation in UC responds to therapeutic interventions that target eicosanoid production such as 5-ASA [13], [14]. However, the understanding of both the pathophysiology of UC and pharmacotherapeutic effects of 5-ASA is limited, which has inhibited the development of new therapeutic interventions.

Eicosanoids are a family of lipid mediators, derived from polyunsaturated fatty acids (PUFA) enzymatically and oxidatively [15]. Previous studies have demonstrated up regulation of the eicosanoid biosynthetic enzymes cyclooxygenase (COX)-1, COX-2, and 5-lipoxygenase (LOX) in active UC [16] and elevation of both prostaglandin (PG)E_2_ [17] and leukotriene (LT)B_4_ [18] derived from the n-6 PUFA arachidonic acid (AA) in UC. Eicosanoids may also derive from the n-3 PUFA eicosapentaenoic acid (EPA); EPA-derived eicosanoids include PGE_3_ and LTB_5_. Frequently the mediators produced from AA and EPA differ in their inflammatory potency [19], [20]. However, studies attempting to alter eicosanoid production through dietary modification of mucosal lipid profile in active UC have been disappointing, with only modest effects on relapse rates and corticosteroid requirement, despite sound experimental results in healthy volunteers [21], [22].

The aims of this study were to investigate the levels of PUFA-derived lipid mediators within inflamed colonic mucosa in mild to moderate UC, to determine their relationship to endoscopic and histological changes, to identify mediators that have not previously been considered possible contributors to the inflammatory cascade in UC, and, as a result, to identify potential targets for intervention. The investigation of patients with distal colitis enabled comparative assessment of endoscopically normal and inflamed tissues as a model, as previously described [2].

## Materials and Methods

### 2.1. Subjects and Mucosal Biopsy

This was a case-control of UC patients identified during attendance at gastroenterology outpatients’ clinics at the Royal Haslar and St Mary’s Hospital, Portsmouth Hospitals NHS Trust between November 2008 and July 2009. The diagnosis of UC was based on endoscopic and histological investigation [23], [24]. All recruited patients underwent a questionnaire-based assessment of their demographic characteristics, previous and presenting medical history, and UC history. Clinical disease activity was determined using the UCDAI score [25].

Patients with clinical evidence of active disease underwent unprepared flexible sigmoidoscopy examination as part of their routine clinical management. An endoscopy assessment was undertaken by two endoscopists blinded to the patient’s clinical presentation using a validated scoring tool to stratify patients into active or quiescent UC with photographic evidence obtained, and to identify demarcation of inflamed with apparently non-inflamed mucosa. Biopsies were taken according to a standard protocol, specifically developed to measure target analytes in adjacent areas of inflamed and non-inflamed mucosa in patients with active distal UC. Flexible sigmoidoscopy in patients with active distal UC to above visual demarcation of normal mucosa was followed by grab biopsy, obtained using flexible biopsy forceps, in endoscopically inflamed and non-inflamed areas. All mucosal biopsy samples were snap-frozen and stored in liquid nitrogen until use.

Exclusion criteria were age less than 16 or greater than 80 years; refusal to undergo endoscopic evaluation; diagnosis of colitis of alternative aetiology; concurrent use of non-steroidal anti-inflammatory medication; inability to provide consent.

The study was approved by the Isle of Wight, Portsmouth and South East Hampshire research ethics committee (project number 08/H0501/82). All subjects provided written informed consent.

### 2.2. Analysis of Lipid Mediators

#### 2.2.1. Reagents and standards

PGE_2_, PGD_2_, thromboxane (TX)B_2_, 6-keto-PGF_1α_, PGB_2_-*d*_4_, 13,14-dihydro-15-keto-PGE_2_, 9-hydroxy-10(*E*),12(*Z*)-octadecadienoic acid (9-HODE), 13-hydroxy-9(*Z*),11(*E*)-octadecadienoic acid (13-HODE), 5-hydroxy-6(*E*),8(*Z*),11(*Z*),14(*Z*)-eicosatetraenoic acid (5-HETE), 8-hydroxy-5(*Z*),9(*E*),11(*Z*),14(*Z*)-eicosatetraenoic acid (8-HETE), 11-hydroxy-5(*Z*),8(*Z*),12(*E*),14(*Z*)-eicosatetraenoic acid (11-HETE), 12(*S*)-hydroxy-5(*Z*),8(*Z*),10(*E*),14(*Z*)-eicosatetraenoic-*5*,*6*,*8*,*9*,*11*,*12*,*14*,*15*-*d*_8_ acid (12-HETE-*d*_8_), 9-hydroxy-5(*Z*),7(*E*),11(*Z*),14(*Z*)-eicosatetraenoic acid (9-HETE), 15-hydroxy-5(*Z*),8(*Z*),11(*Z*),13(*E*)-eicosatetraenoic acid (15-HETE), 12-hydroxy-5(*Z*),8(*Z*),10(*E*),14(*Z*)-eicosatetraenoic acid (12-HETE) and LTB_4_ standards were purchased from Cayman Chemicals (Ann Arbor, MI). HPLC-grade acetonitrile, ethanol, methanol, hexane, and hydrochloric acid were from Fisher Chemicals (Loughborough, UK), HPLC-grade acetic acid and methyl formate from Sigma (Dorset, UK), and solid-phase extraction (SPE) cartridges (C_18_-E, 500 mg, 6 mL) from Phenomenex (Macclesfield, UK).

#### 2.2.2. Solid phase extraction

Extraction of lipid mediators was carried out according to a previously described method [26]. In summary, mucosal biopsy samples (approximately 3–5 mg) were weighed on thawing and immediately transferred to 15% ice-cold methanol in water (3 mL) and homogenised using Wheaton Tapered tissue grinders. Internal standards (PGE_2_-*d*_4_ and 12-HETE-*d*_8_ (20 ng)) were added to each sample. The samples were acidified with 0.05 M hydrochloric acid to pH 3.0 and immediately applied to SPE cartridges that had been preconditioned with 20 mL of methanol, followed by 20 mL of water. The cartridges were then washed with 20 mL of 15% (v/v) methanol, 20 mL of water, and 10 mL of hexane. Finally, the lipid mediators were eluted with 10 mL of methyl formate. The organic solvent was evaporated using a fine stream of nitrogen, the solute reconstituted in ethanol (100 µL), and the final solution stored at −20°C until analysis within 48 hours.

#### 2.2.3. Lipidomics analysis

Data-dependent LC-MS/MS lipidomics analysis was performed using Accela UHPLC system (Thermo Scientific, Hemel Hempstead, UK) coupled to LTQ Velos (Thermo, Hemel Hempstead, UK) linear ion trap (LIT)-orbitrap as described previously [26]. The analysis on the orbitrap instrument was carried out using heated electrospray ionization (h-ESI) in negative ion mode at sheath, auxiliary and sweep gas flows of 24, 2 and 1, respectively [26]. The capillary and source heater temperatures were set to 275°C and 50°C, respectively. The ion spray voltage was adjusted to 4000 V. MS/MS spectra, along with retention times and isotope distribution patterns from the MS spectra were used to identify lipid mediators in mucosal biopsy samples. The identified metabolites were quantified using Acquity UPLC (Waters, Hertsfordshire, UK) systems coupled to QTRAP 4000 (AB Sciex, Concord, ON) quadrupole−linear ion trap (QqLIT) mass spectrometers as described previously [26]. The lipid mediators were separated on a C_18_ reversed-phase (RP) LC column (Phenomenex Luna, 3 µm particles, 150×2 mm) using a linear mobile phase gradient (A, 0.02% glacial acetic acid in water; B, 0.02% glacial acetic acid in acetonitrile) at 0.5 mL/min. The starting conditions consisted of 30% B and then increased to 90% B over 14 min and finally returned to the initial conditions for 2 min to allow equilibration.

### 2.3. Histological Analysis

**Figure 1:**
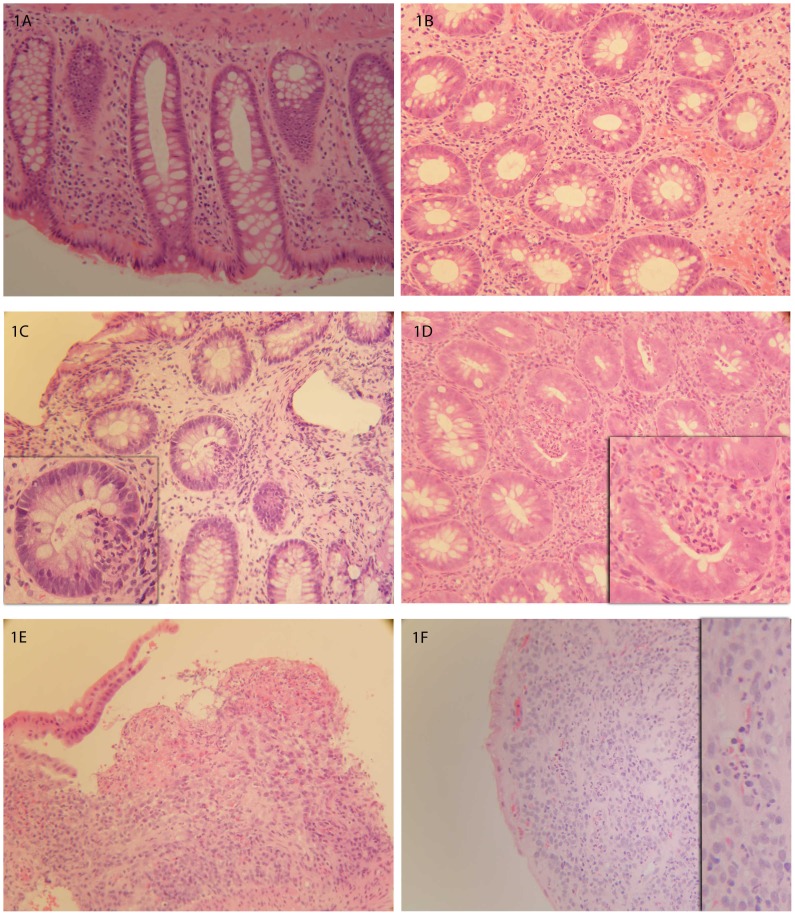
Gomes histological classification of UC. 1A: Gomes 0 (normal); 1B: Gomes 1 (mild oedema and inflammation in lamina propria with cryptitis); 1C: Gomes 2 (crypt abscess formation and inflammation); 1D: Gomes 3 (destructive crypt abscesses +/− granulomata); 1E–F: Gomes 4 (active ulceration and formation of granulation tissue with neoangiogenesis).

Assessment was by a histopathologist blinded to patients’ identities and clinical data on paraffin embedded serial haematoxylin and eosin (H&E) stained sections using a validated histopathological scoring tool [24] (0, normal; 1, mild oedema and inflammation in lamina propria; 2, crypt abscess formation and inflammation in lamina propria; 3, more severe inflammation with destructive crypt abscesses +/−granulomata; 4, more severe inflammation with active ulceration) (**Figure 1**).

### 2.4. Statistical Analysis

Lipid mediator concentration is expressed as pg/mg tissue. Unblinded bioinformatics analysis was carried out following completion of lipid mediator measurement on blinded samples.

#### 2.4.1. Univariate statistical analysis

Wilcoxon’s Signed Rank Test was used as a non-parametric pair-wise univariate testing method at a confidence interval of 97.5%. Analysis was carried out using custom workflows written in the programming language R. Metabolites with a value of p<0.05 were regarded as significantly different. Subgroup analysis to investigate the effect of no 5-ASA or corticosteroid (*n*18) use was undertaken and metabolites with a value of p<0.05 were regarded as significantly different.

#### 2.4.2. Multivariate statistical analysis

Since univariate statistical testing is unable to characterise the interrelationship of variables, multivariate statistical analysis was performed to examine association with disease state (inflamed/non-inflamed) as well as disease severity assessed histologically using the Gomes scoring system. Similar to the paired univariate testing, we were primarily interested in the within-subject variation and therefore performed a variation split prior to analysis (to remove between subject variation), a technique developed in the context of multi-level multivariate data analysis [27], [28].

On the remaining within-subject variation data we performed Principal Component Analysis (PCA) for unsupervised analysis, followed by (Orthogonal) Partial Least Squares (PLS) Analysis for the association with disease severity assessed histologically, and (Orthogonal) Partial Least Squares Discriminant Analysis (PLS-DA) for the investigation of the disease state (inflamed/non-inflamed). Variation splitting was implemented using the programming language R, Partial Least Squares Approaches were performed using R as well as SIMCA-P 12.01 (Umetrics, AB, Sweden).

## Results

### 3.1. Subjects

Patients with active UC (*n* 69) were recruited; biopsy data sets of ‘paired’ macroscopically inflamed and non-inflamed tissues were available in 54 UC patients. Baseline characteristics of patients are shown in **Table 1**.

**Table 1:** Baseline characteristics of patients with active UC.

Category	Subcategory	Result
Sex [n(%)]	Male	26 (48)
	Female	28 (52)
Age [yr]∞		44.4±1.8
CRP [mg/l]∞		7.0±1.1
BMI [kg/m^2^]∞		27.5±0.8
Disease Distribution [n(%)]	Proctitis	32 (60)
	Distal	16 (30)
	Left sided	3 (6)
	Extensive	3 (6)
Disease Longevity [yr(%)]	0–5	22 (41)
	5–10	15 (28)
	10–15	6 (11)
	15–20	3 (6)
	>20	7 (13)
	unknown	1 (2)
Current drug history [n(%)]	5-ASA	32 (60)
	Corticosteroids	14 (26)
	Thiopurines	8 (15)
	Methotrexate	1 (2)
	Cyclosporine	1 (2)
Disease activity-UCDAI* ^,^ ∞		4.8±0.4
Disease activity-endoscopicscore [n(%)]¥	1	36 (67)
	2	13 (24)
	3	5 (9)
Disease activity-histologicalscore [n(%)]€	0	8 (15)
	1	24 (44)
	2	11 (20)
	3	8 (15)
	4	3 (6)
Smoking history [n(%)]	yes	6 (11)
	no	48 (89)

* UCDAI-ulcerative colitis disease activity index [59] (Daily stool frequency [0 = normal; 1 = 1–2 above normal; 2 = 3–4 above normal; 3 = >4 above normal]+Rectal bleeding [0 = none; 1 = streaks of blood; 2 = obvious blood; 3 = mostly blood]+Sutherland score [see below]+Physicians global assessment [0 = normal; 1 = mild; 2 = moderate; 3 = severe]).

¥ Sutherland endoscopic scoring tool [25] (0 = normal; 1 = mild friability; 2 = moderate friability, bleeding on contact; 3 = exudation, spontaneous haemorrhage).

€ Gomes Histological score [24] (0 = normal, 1 = mild oedema and inflammation in lamina propria; 2 = crypt abscess formation and inflammation in lamina propria; 3 = more severe inflammation with destructive crypt abscesses +/−granulomata; 4 = more severe inflammation with active ulceration).

∞ mean ± SEM.

### 3.2. Lipid Mediators: Univariate Analysis

Mucosal biopsies from inflamed and non-inflamed mucosa were screened for all PUFA-derived bioactive lipids and deactivated metabolites. Reported metabolites included eicosanoids derived from AA (TXB_2_, 6-keto-PGF_1α_, PGE_2_, PGD_2_, 5-HETE, 15-HETE, 12-HETE and 11-HETE) and linoleic acid (LA) (13-HODE, 13-oxo-ODE, 9-HODE). EPA-derived eicosanoids (5-hydroxy-eicosapentaenoic acid (HEPE), 12-HEPE, 15-HEPE) were excluded from analysis as their concentration was below the limit of quantification. Neither PGE_3_ nor LTB_5_ were detected.

#### 3.2.1. LOX-related lipid mediators

**Figure 2:**
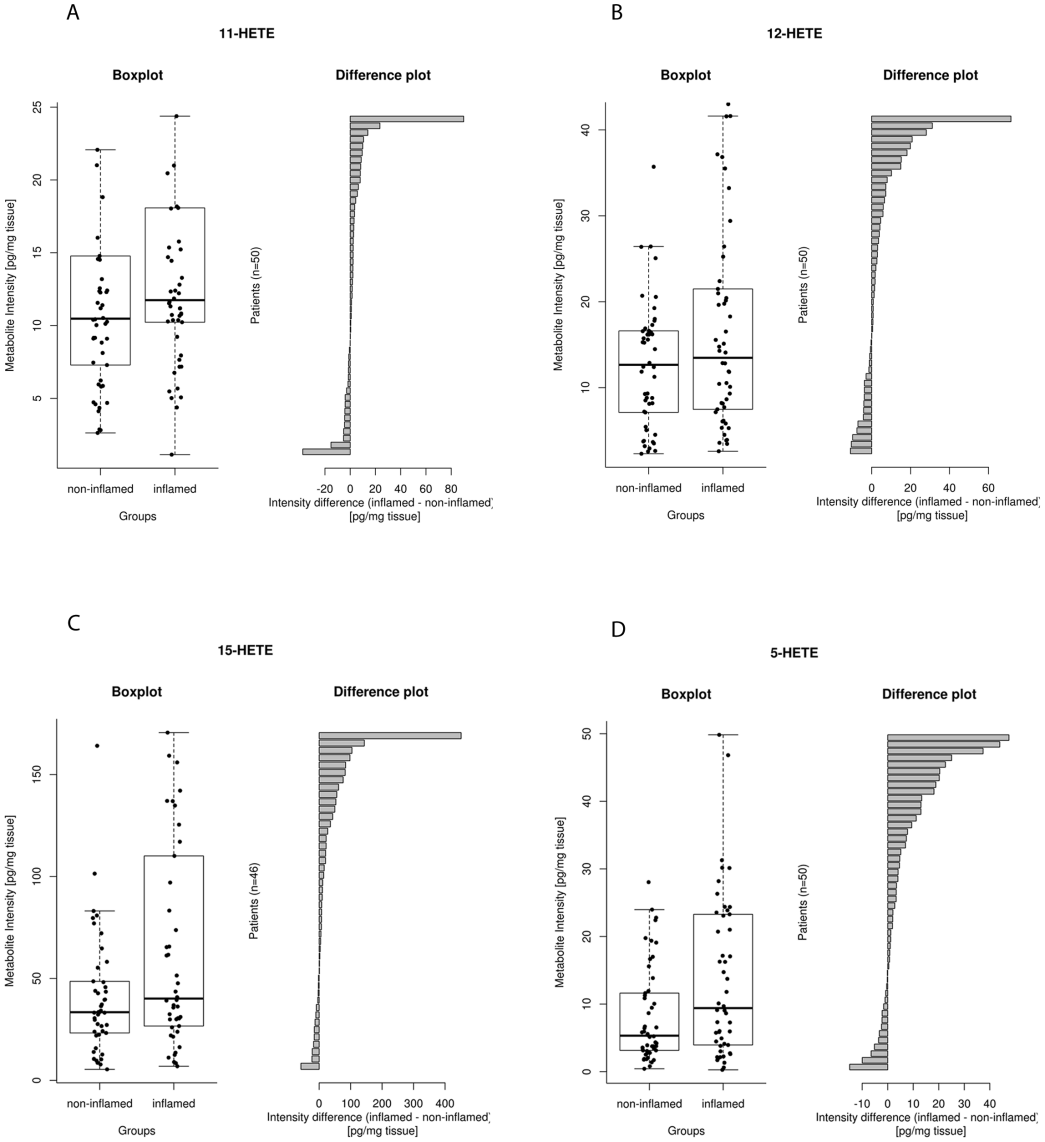
Median and interquartile range of LOX-related lipid mediators (pg/mg tissue) in grouped non-inflamed and adjacent inflamed mucosa with difference plots of paired samples. 2A: 11-HETE; 2B: 12-HETE; 2C: 15-HETE; 2D: 5-HETE.

5-HETE (p<0.001) and 15-HETE (p<0.001) concentrations in inflamed mucosa were significantly higher than in adjacent non-inflamed mucosa (Table 2, Figure 2). Other 5-LOX metabolites of AA such as LTB_4_ and 5-oxo-ETE were inconsistently detected. There were no significant differences in the concentrations of LA-derived LOX metabolites such as 9-HODE, 13-HODE and 13-oxo-ODE.

**Table 2:** Lipid mediator concentrations (pg/mg tissue) in colonic mucosa (inflamed and non-inflamed) in UC patients.

	Paired data		
Lipid mediator	Inflamed€	Non-inflamed€	p-value*
TXB_2_	46.2 (27.2–70.7)	32.2 (20.6–41.7)	<10^−6^
6-keto-PGF_1α_	34.9 (17.9–51.4)	29.5 (14.8–48.4)	0.201
PGE_2_	24.1 (13.4–56.2)	9.0 (4.8–15.3)	<10^−6^
PGD_2_	13.9 (5.9–21.9)	12.1 (2.5–16.4)	0.001
9-HODE	27.4 (20.1–45.7)	33.9 (19.5–42.3)	0.368
5-HETE	9.4 (4.0–23.2)	5.3 (3.2–11.5)	<10^−3^
15-HETE	39.2 (26.0–97.0)	33.4 (23.4–48.5)	<10^−3^
13-oxo-ODE	30.1 (19.1–42.0)	31.0 (21.6–42.2)	0.320
13-HODE	57.1 (30.9–91.4)	67.1 (31.9–88.8)	0.650
12-HETE	13.5 (7.5–21.4)	12.7 (7.1–16.6)	0.021
11-HETE	11.7 (10.2–18.1)	10.5 (7.3–14.7)	0.028

€ Data are median ± IQR.

* Wilcoxon signed rank pair analysis.

#### 3.2.2. COX-related lipid mediators

**Figure 3:**
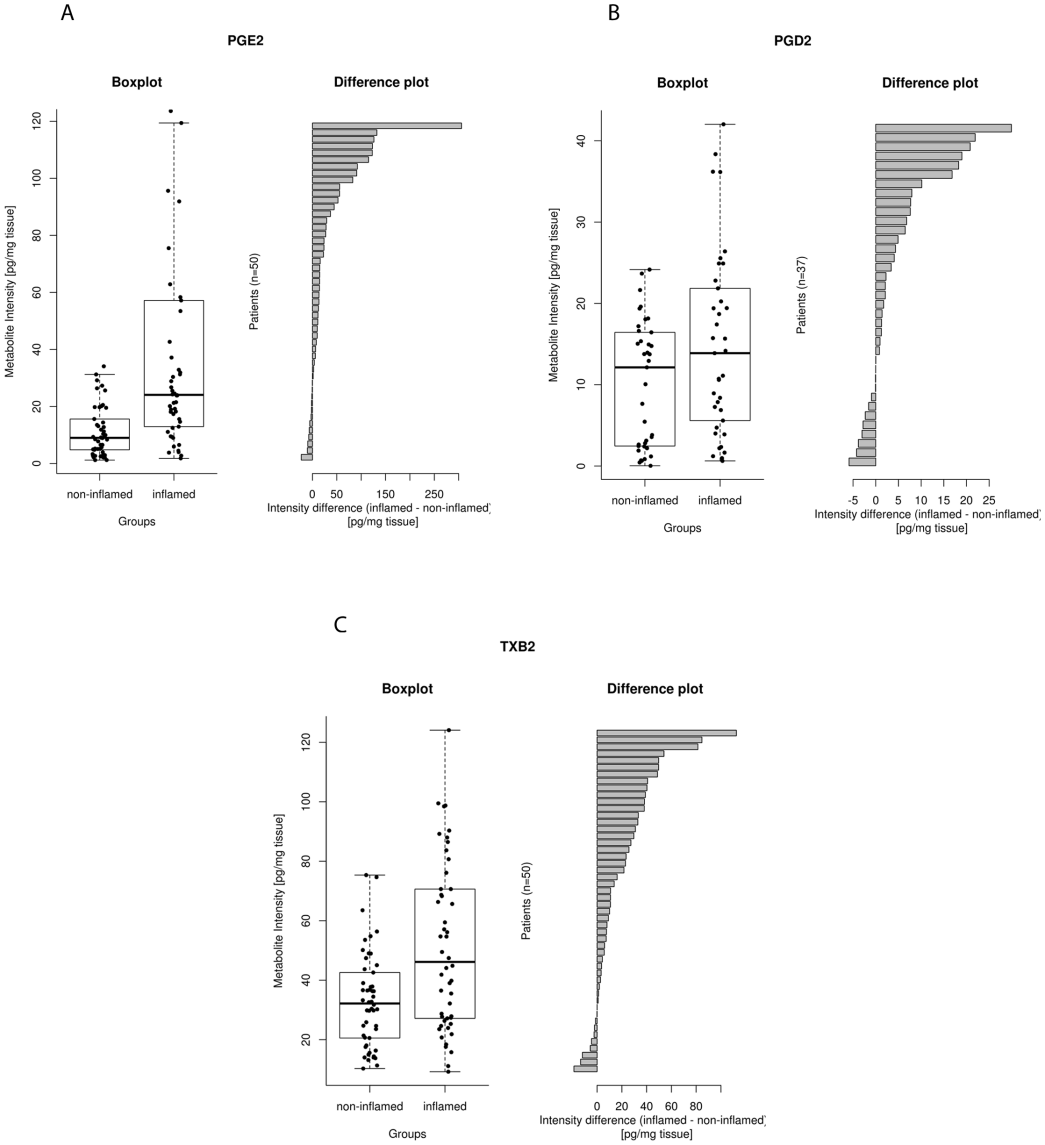
Median and interquartile range of COX-related lipid mediators (pg/mg tissue) in grouped non-inflamed and adjacent inflamed mucosa with difference plots of paired samples. 3A: PGE_2_; 3B: PGD_2_; 3C: TXB_2_.

PGE_2_ (p<10^−6^), PGD_2_ (p<0.01) and TXB_2_ (p<10^−6^) concentrations in inflamed mucosa were significantly higher than in adjacent non-inflamed mucosa (Table 2; Figure 3). There were no other significant differences in concentration of COX metabolites between non-inflamed and inflamed mucosa. PGF_2α_ and the deactivated form of PGE_2_, 13,14-dihydro-15-keto-PGE_2_, were only detectable in 50% of the samples and therefore were excluded from analysis.

### 3.3. Lipid Mediators: Multivariate Analysis

**Figure 4:**
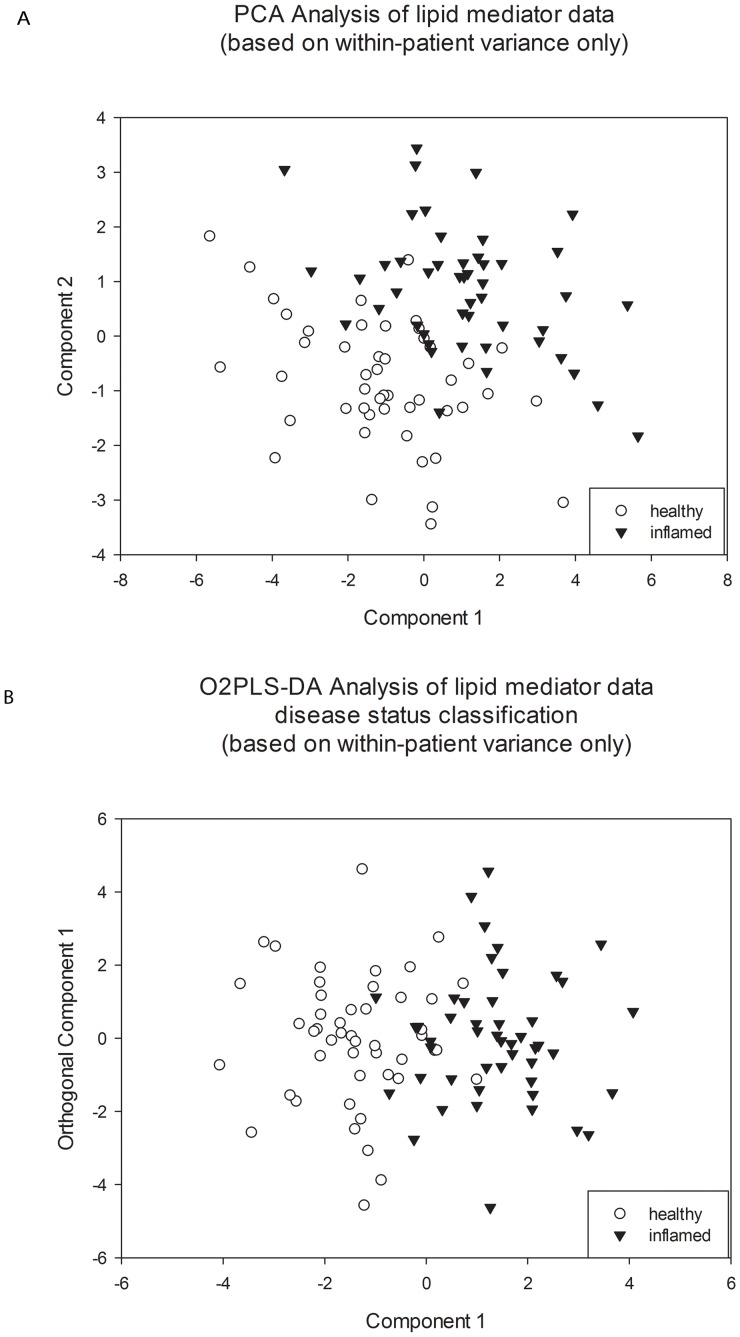
Data separation of inflamed (triangle) and non-inflamed (circle) mucosa based on lipid mediator concentration. 4A: PCA score plot (unsupervised analysis); 4B: O2PLS-DA plot (supervised analysis).

The principal component analysis (PCA), an unsupervised multivariate analysis technique, which is driven by the variance inherent in the data set and has no prior assumption of class membership, revealed a clear differentiation between inflamed and endoscopically non-inflamed adjacent mucosal tissue in patients with distal UC (Figure 4A).

Furthermore, O2-PLSDA, a supervised analysis, was performed to actively check for differences in the lipid mediator profile between inflamed and non-inflamed tissue. As expected from the PCA analysis, the O2PLS-DA model (R^2^X(cum) = 79%, R^2^Y(cum) = 60% and Q^2^ = 60%) was clearly able to differentiate the two phenotypic groups (**Figure 4B**). Using the O2PLS-DA model, 87.5% of inflamed tissue was correctly identified as inflamed based upon the lipid mediator profile, while 87.5% of non-inflamed tissue was correctly identified as non-inflamed.

**Figure 5:**
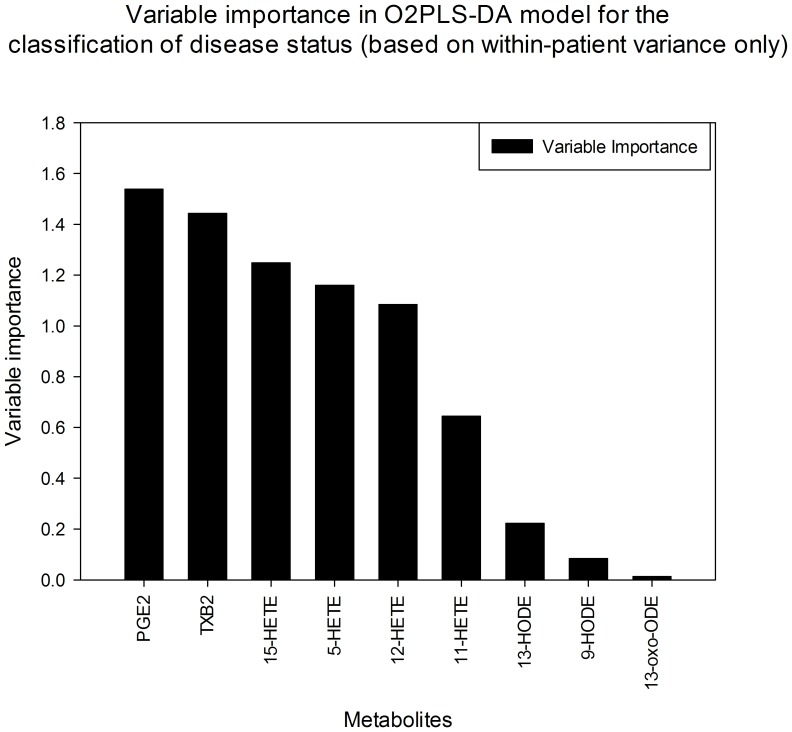
Variable importance plot in O2PLS-DA analysis demonstrates relative contribution of lipid mediators to separation between inflamed and non-inflamed mucosa. (Fig. 4).

In order to investigate which metabolites made the greatest contribution to the separation observed in O2PLS-DA model (**Figure 4B**), a variable importance plot was created; PGE_2_ had the highest contribution followed by TXB_2_, 15-HETE, 5-HETE, 12-HETE and 11-HETE, respectively in diminishing magnitude (**Figure 5**). This is consistent with the results of the univariate statistical analysis (**Table 2**), and demonstrates contribution of 12-HETE and 11-HETE which were significantly higher (all p<0.05) in inflamed compared with non-inflamed colonic mucosa in the univariate analysis. PGD_2_ was excluded from this multivariate analysis as it was detected in <85% of samples.

### 3.4. Association between Lipid Mediators and Clinical Grading

**Figure 6:**
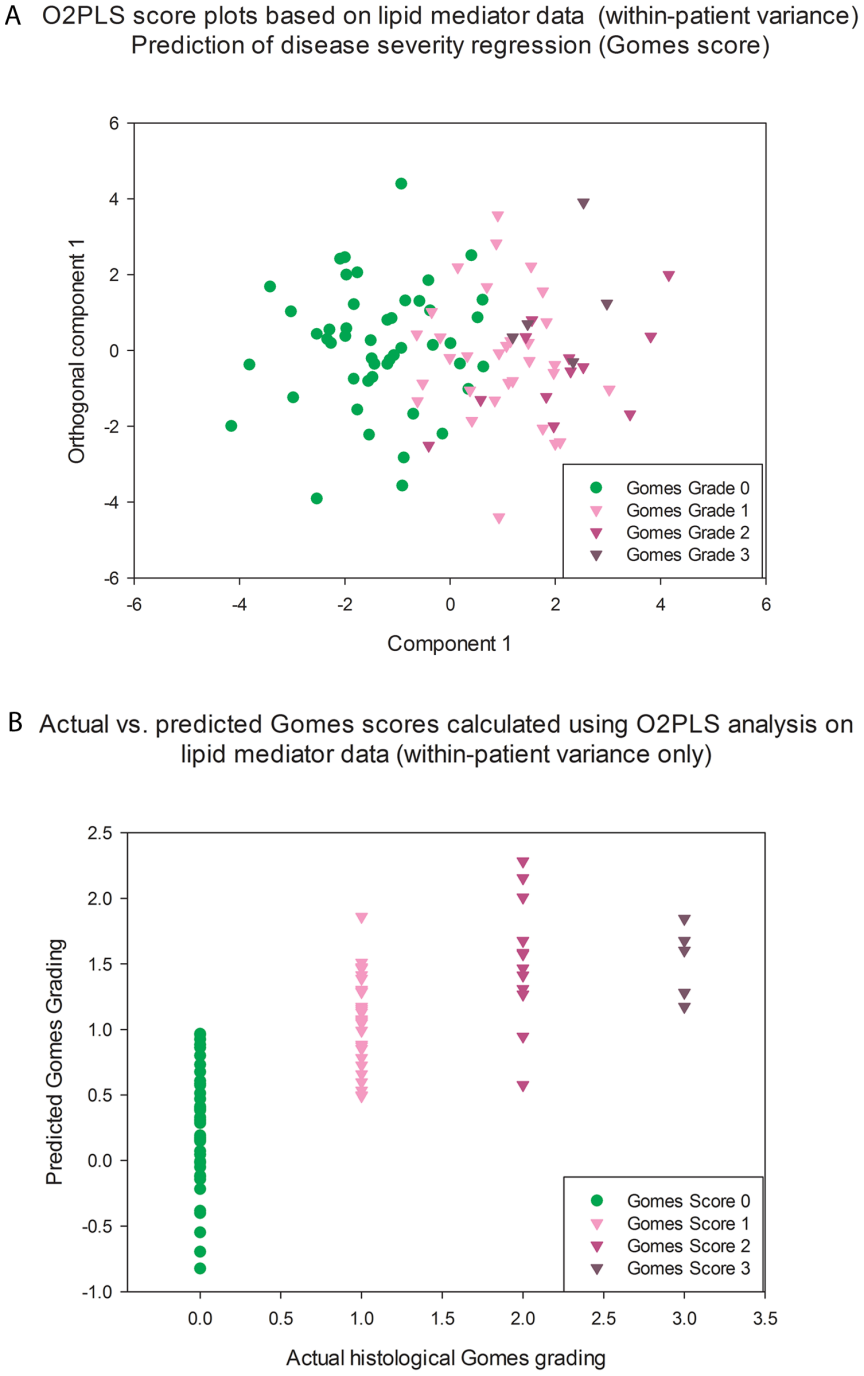
O2PLS model showing computed prediction of disease severity based on lipid mediator profile. 6A: Plot showing O2PLS regression score plots; 6B: plot showing actual vs. predicted GOMES score based on lipid mediator profile.

O2-PLS Analysis was performed in order to examine the relationship between lipid mediator concentration and histological grading (**Figure 6**). The model (R^2^X(cum) = 86%, R^2^Y(cum) = 57% and Q^2^ = 51%) was able to separate non-inflamed from inflamed tissue; however within the inflamed tissue there was an overlap between histological grades (**Figure 6A**). Although it was possible to predict mild-moderate inflammation (grade 1 and 2) from the lipid mediator profile (**Figure 6B**), highly inflamed tissue (grade 3), failed to fit into this model; however this latter group contained 5 subjects only.

**Figure 7:**
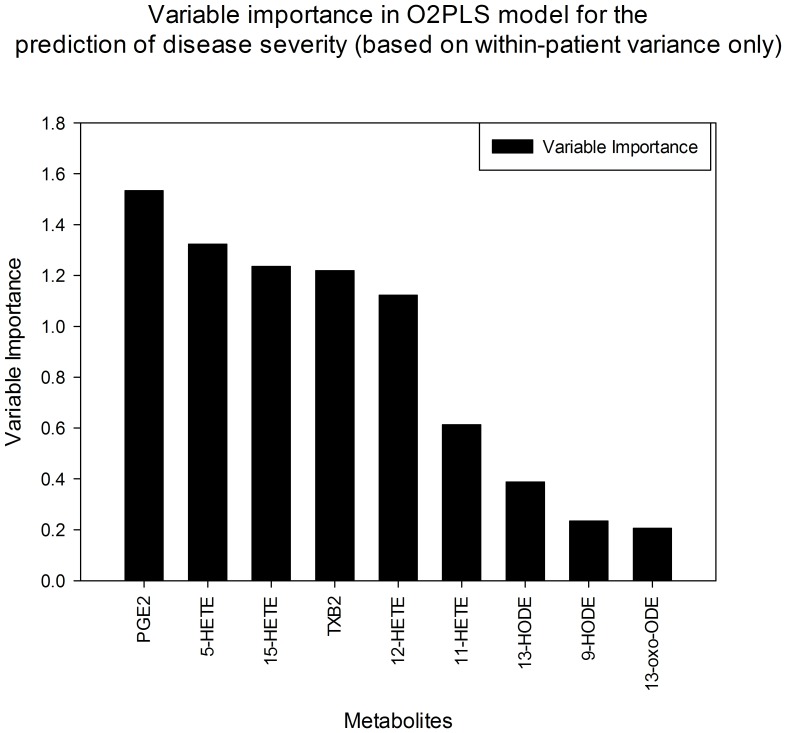
Variable importance plot in O2PLS analysis demonstrates relative contribution of lipid mediators to separation between histological grades of severity. (Fig. 6).

In order to investigate which metabolites had the greatest contribution to the separation observed in the O2PLS model, a variable importance plot was created; PGE_2_ showed the highest impact followed by 5-HETE, 15-HETE, TXB_2_, 12-HETE and 11-HETE, respectively in diminishing magnitude (**Figure 7**).

### 3.5. 5-ASA and Corticosteroid Naive Patients

Subgroup analysis within ‘paired’ inflamed and non-inflamed mucosa from 18 UC patients (age 45.3 years; male *n* 10 [55%], female *n* 8 [45%]) not recently (within 3 months) on 5-ASA or corticosteroid treatment was carried out. Analysis of lipid mediators demonstrated that PGE_2_, PGD_2_, TXB_2_ and 15-HETE (all p<0.05) were higher in inflamed than non-inflamed mucosa; however, the other measured lipid mediators were not significantly different (**Table 3**). The results within the subgroup analysis are comparable to the total group univariate analysis.

**Table 3:** Lipid mediator concentrations (pg/mg tissue) in colonic mucosa (inflamed and non-inflamed) in treatment naive UC.

	Paired data		
Lipid mediator	Inflamed€	Non-inflamed€	p-value*
TXB_2_	43.4 (27.6–59.3)	28.7 (15.9–48.1)	0.002
6-keto-PGF_1α_	34.9 (25.2–51.5)	37.9 (5.8–47.4)	0.776
PGE_2_	27.1 (16.9–42.8)	7.1 (2.4–15.9)	<10^−3^
PGD_2_	10.3 (5.4–23.4)	3.8 (2.2–15.4)	0.016
9-HODE	25.2 (15.0–46.8)	30.6 (17.6–55.1)	0.879
5-HETE	14.7 (7.0–23.4)	10.4 (4.6–19.4)	0.191
15-HETE	40.8 (23.3–103.6)	39.1 (13.6–51.0)	0.005
13-oxo-ODE	24.4 (15.1–37.1)	31.0 (13.7–43.6)	0.913
13-HODE	60.8 (28.4–96.0)	67.5 (29.2–95.4)	0.811
12-HETE	14.7 (7.7–25.2)	13.9 (5.3–20.6)	0.133
11-HETE	10.0 (6.7–13.6)	9.1 (5.1–10.7)	0.053

€ Data are median ± IQR.

* Wilcoxon signed rank pair analysis.

### 3.6. Controls

Lipid mediator levels (TXB_2_ – 38.8 [27.5–51.6]; 6-keto-PGF_1α_ – 36.1 [27.8–49.5]; PGE_2_ – 14.9 [7.1–24.1]; PGD_2_ – 9.9 [4.0–17.7]; 9-HODE – 38.3 [20.0–55.6]; 5-HETE – 6.2 [3.0–12.4]; 15-HETE – 19.1 [10.7–32.4]; 13-oxo-ODE – 21.6 [14.8–41.3]; 13-HODE – 55.4 [29.8–84.2]; 12-HETE – 9.1 [5.5–16.5]; 11-HETE – 9.4 [7.0–15.7]) were measured in normal colorectal mucosa obtained from a group of 42 control patients undergoing routine flexible sigmoidoscopy examination.

## Discussion

In the current study we demonstrate differences in the concentration of a number of lipid mediators between inflamed and non-inflamed areas of the colonic mucosa from patients with UC. The findings suggest that inflammation of the colonic mucosa in UC is associated with significant elevation in concentrations of PGE_2_, PGD_2_, TXB_2_, 5-HETE, 15-HETE, 12-HETE and 11-HETE, but not of other measured lipid mediators (**Table 2**). In addition, the profile of these same lipid mediators correlates with severity of inflammation measured histologically. To our knowledge this is the first study to simultaneously demonstrate comprehensive alterations in multiple lipid mediators, which correlate proportionately to the degree of histological inflammation in patients with UC. This is also in agreement with previous studies that showed upregulation in eicosanoids, which correlate proportionately to the degree of histological inflammation in patients with UC; however, previous studies have been limited to investigation of selected enzymatic pathways (COX-2 and 5-LOX) in UC[16]–[18], [29]–[32].

Several of the identified inflammatory mediators have immunomodulatory roles as demonstrated either in cohorts of inflammatory bowel disease (IBD) patients or in experimental studies. For example, PGE_2_ is produced via COX-1 and COX-2 within the AA cascade and has pro-inflammatory (via cytokine induction pathways) and anti-inflammatory (via lipoxin induction) effects [33], [34]. PGE_2_ has been consistently demonstrated in previous studies to be elevated in inflamed colonic mucosa in UC [17]. 12-HETE is produced via 12-LOX within the AA cascade and is known to exert chemotactic effects on neutrophils [35], [36]. 5-HETE and LTB_4_ are both products of 5-LOX within the AA cascade; neutrophils contain large quantities of 5-LOX and are able to produce abundant 5-HETE and LTB_4_ [37], [38]. 5-HETE is a potent activator of eosinophils and neutrophils via 5-oxo-6,8,11,14-eicosatetraenoic acid (5-oxo-ETE). 5-HETE and 12-HETE were readily detected in inflamed mucosal samples.

LTB_4_ is a potent chemoattractant for neutrophils and other leukcoytes [39]; however, LTB_4_ was not consistently demonstrated in our samples. LTB_4_ is reported to be elevated in inflamed mucosa in UC; however, critical review of published methods reveals that levels were measured in *in vivo* rectal dialysates or mucosal explants, which would have led to neutrophil activation inadvertently or by design [40]–[48]. The presented method controls for altered lipid mediator production induced by experimental methods. The lack of consistent demonstration of LTB_4_ in the context of elevated 5-HETE (both 5-HPETE derivatives) may suggest that the bioactive concentration of LTB_4_ is below the detectable limit, or that it is rapidly metabolised in inflamed mucosal samples. An alternative explanation, that 5-HETE is the predominant 5-LOX derived chemoattractant lipid mediator, or that PGE_2_ mediated inhibition of FLAP (5-lipoxygenase activating protein) abrogates LTB_4_ production, should be considered [49]; however, this was not specifically tested in our study.

The pro-inflammatory mediator TXB_2_, a stable derivative of TXA_2_ and both AA derivatives via COX-dependent conversion from PGH_2_, is elevated in inflamed compared with non-inflamed mucosa, as has been demonstrated previously [50].

In contrast, PGD_2_ and its metabolite 15-deoxy Δ^12,14^ PGJ_2_ exert anti-inflammatory effects [51], and several studies have suggested a role for PGD_2_ in resolution of inflammation, reduction of leukocyte infiltration and healing within the colon[32], [52]–[54]. Consistent with other studies, we observed a significant increase in the levels of PGD_2_ in inflamed tissue, although its metabolite 15-deoxy Δ^12,14^ PGJ_2_ was not detectable in mucosal biopsies.

Sub-group analysis (*n* 18) of a group of patients not receiving corticosteroids and 5-ASA confirmed significant differences in PGE_2_, PGD_2_, TXB_2_ and 15-HETE in inflamed mucosa (**Table 3**), with no significant differences observed in 5-HETE, 11-HETE and 12-HETE concentrations in inflamed compared with non-inflamed mucosa. This may indicate the diminished power to detect differences in all measured eicosanoids in this group due to sample size, or possibly a more benign clinical course in this untreated group.

Using predictive mathematical modelling, we have demonstrated that the measured lipid mediator profile may be used to predict presence or absence of histological inflammation with sensitivity, specificity, and positive predictive and negative predictive values of 87.5%. It was also possible to grade severity of inflammation based on blinded histological assessment; however, predictive modelling was less consistent in patients with severe inflammation. This may be due to the small group of patients (*n* 5) in this group, or may suggest that alternative inflammatory mediators become predominant in severe mucosal inflammation.

Eicosanoids and other lipid mediators are generated via oxidative pathways (COX and LOX) at the cytosolic interface of the cell membrane. They have pleiotropic effects and biochemical activity is influenced by the species of PUFA from which they are derived. In excess of 40 different eicosanoids from PUFA substrates are recognised. These are derived from obligate dietary constituents, LA, AA and EPA, but principally from AA due to the higher membrane content of AA and the lower bioavailability of EPA. Provision of EPA orally can influence the levels of AA in cell membranes and reduce pro-inflammatory AA derived lipid mediators in patients with IBD [55] and in healthy subjects [56]; although clinical therapeutic efficacy is inconsistently demonstrated [21], [57].

In the current study, although AA-derived lipid mediators (PGE_2_, PGD_2_, TXB_2_, 5-HETE, 15-HETE, 12-HETE and 11-HETE) were detectable in inflamed mucosa, EPA-derived mediators (PGE_3_, PGD_3_, TXB_3_, 5-HEPE, 15-HEPE, 12-HEPE and 11-HEPE) were not, or were detected at unquantifiable levels. We previously reported that actively inflamed mucosa in UC is associated with increased AA and reduced EPA in both the complex lipid and free fatty acid pools, with a consistently altered AA/EPA ratio compared with adjacent non-inflamed mucosa, and that this alteration is despite no significant difference in the level of dietary intake of PUFA (Pearl et al) [42]. These findings are consistent and suggest that metabolic alteration of AA production in inflamed tissue may lead to enhanced substrate availability for eicosanoid biosynthetic enzymes which are constitutively up regulated in actively inflamed mucosa, with resulting up regulation in all classes of eicosanoids, which we have detected.

These findings may be relevant to the failure of dietary intervention studies with EPA. This may be because EPA and its lipid mediators including eicosanoids are present at very low levels in inflamed mucosa, whereas AA and its lipid mediators including eicosanoids are present at much higher levels. Supplementation with EPA may not bring about significant rebalancing of the AA/EPA ratio, which would be required to reduce the levels of AA derived eicosanoid lipid mediators in inflamed mucosa.

In addition, eicosanoids derived from both COX- and LOX-related pathways are significantly elevated indicating co-activation of multiple eicosanoid biosynthetic pathways within the AA cascade in inflamed mucosa. The role of eicosanoids is further supported by the known therapeutic effect of 5-ASA drugs, which act by binding to peroxisome proliferator activated receptor γ in the colonic epithelium with subsequent suppression of pro-inflammatory lipid mediator production [18].

Co-activation of multiple synthetic pathways may suggest redundancy within eicosanoid signalling networks; however, an alternative explanation is that candidate eicosanoid-receptor signalling independently stimulates initiation, propagation, resolution and/or suppression stages of inflammation. This is supported by the observed loss of intestinal eicosanoid homeostasis, as occurs with COX inhibition during non-steroidal anti-inflammatory drug use, which is associated with an increased risk of UC relapse in quiescent disease [58]. This is also supported by detection of pro-inflammatory (PGE_2_) and anti-inflammatory (PGD_2_) lipid mediators within the same tissue matrix, as in the current study [51]_._

The role of detected eicosanoids (PGE_2_, PGD_2_, TXB_2_, 5-HETE, 15-HETE, 12-HETE and 11-HETE) in UC is difficult to address precisely, as this study was not designed to investigate changes occurring as a result of therapy, or temporal changes in mucosal lipid mediators associated with disease activity.

There is therefore emergent evidence that UC is associated with changes in PUFA profile with elevated AA/EPA ratio suggesting alteration of production of the eicosanoid substrate AA (Pearl et al) [42], and with associated elevation of AA-derived eicosanoids which correlate with grade of histological inflammation.

Further research should focus on the use of predictive modelling based on mucosal derived lipid mediators to determine if drug non-responders can be predicted, and to target appropriate therapy. This would be a unique development in management of chronic inflammatory disorders.

Additional research could focus on characterization of lipid mediators biosynthesis during initiation, propagation, resolution and suppression stages of inflammation, and to relate these to metabolic activity within the fatty acid biosynthetic cascade. This would allow a focussed time-course assessment of the role of lipid mediators during the evolution of inflammation using distal UC as a model and may inform new targets for development of therapeutic interventions in UC and inflammatory disorders.

In conclusion, development of new approaches and treatments, based on selective lipid mediators, may offer new investigative and therapeutic strategies to target treatment in patients with mild-moderate chronic active steroid and immunomodulator resistant UC in whom an organ sacrificing approach via surgical colectomy is being considered.
